# New Strategy for Inducing Resistance against Bacterial Wilt Disease Using an Avirulent Strain of *Ralstonia solanacearum*

**DOI:** 10.3390/microorganisms10091814

**Published:** 2022-09-10

**Authors:** Zeiad Moussa, Ehsan M. Rashad, Elsherbiny A. Elsherbiny, Abdulaziz A. Al-Askar, Amr Abker Arishi, Fatimah O. Al-Otibi, WesamEldin I. A. Saber

**Affiliations:** 1 Microbial Activity Unit, Microbiology Department, Soils, Water and Environment Research Institute, Agricultural Research Center, Giza 12619, Egypt; 2 Department of Seed Pathology Research, Plant Pathology Research Institute, Agricultural Research Center, Giza 12619, Egypt; 3 Plant Pathology Department, Faculty of Agriculture, Mansoura University, Mansoura 35516, Egypt; 4 Department of Botany and Microbiology, Faculty of Science, King Saud University, Riyadh 11451, Saudi Arabia; 5 School of Molecular Sciences, The University of Western Australia, Perth, WA 6009, Australia

**Keywords:** biological control, bacterial supernatant, defense-related enzymes, GC–MS analysis, lytic enzymes

## Abstract

*Ralstonia solanacearum* is one of the globally significant plant pathogens that infect a wide host range of economically important plants. A study was conducted to evaluate the hypothesis that an avirulent strain of *R. solanacearum* can act as a biocontrol mediator for managing potato bacterial wilt. Virulent *R. solanacearum* was isolated and identified (GenBank accession number; OP180100). The avirulent strain was obtained from the virulent strain through storage for 3 weeks until the development of deep red colonies. The virulent strain had higher lytic activity than the avirulent strain. Tubers’ treatments by the avirulent strain of *R. solanacearum*, (supernatant, boiled supernatant, and dead cells) significantly reduced plant disease rating and increased the growth, physiological activities, and biomass of potato compared to the untreated, infected control. The major components detected by GC–MS in the supernatant revealed 10.86% palmitic acid (virulent), and 18.03% 1,3-dioxolane, 2,4,5-trimethyl- (avirulent), whereas the major component in the boiled supernatant was 2-hydroxy-gamma-butyrolactone in the virulent (21.17%) and avirulent (27.78%) strains. This is the first research that assessed the influence of boiled supernatant and dead cells of virulent and avirulent *R.* *solanacearum* strains in controlling bacterial wilt disease. Additional work is encouraged for further elucidation of such a topic.

## 1. Introduction

*Ralstonia solanacearum* is a unique, important worldwide bacterial plant pathogen according to its economic and scientific value [1], representing the causative pathogen of wilt disease in about 250 species, including essential economic crops, e.g., potato, tomato, banana, and peanut [2]. Potato wilt (brown rot) generated by *R. solanacearum* is a serious disease worldwide, leading to severe yield losses, reaching up to 100% in Kenya [3] and up to 75% in Australia [4]. In Egypt, bacterial wilt was announced many years ago as the most significant bacterial disease on potatoes [5]. The accumulation of bacterial cells, together with the slimy exopolysaccharides, and virulence-related factors lead to blocking the vessels, consequently stopping the sap flow, which in turn, generates the wilt disease [6]. Many chemical, biological, and physical tools were used to control this bacterium. Biological control agents are hopeful eco-friendly tools alternative to pesticides to manage plant diseases that are beneficial in protecting crop plants and safeguarding food as well [7].

The use of the avirulent *R. solanacearum* strain to manage the virulent strain is one of the promising biocontrol strategies [8]. However, studies in such areas are still lacking the best application method. An avirulent strain did not antagonize the virulent strain and was not bacteriocinogenic, but can reduce the disease severity by up to 50% [9,10]. For instance, the bacteriocinogenic avirulent strain of *R. solanacearum* was effectively controlled on tobacco [11] and tomato [12] bacterial wilt. While, on the opposite side, other studies revealed that the avirulent strains of *R. solanacearum* have an antagonistic action versus the virulent *R. solanacearum* strain in vitro and in vivo [9,10]. Furthermore, the avirulent strain of *R. solanacearum* can be used solely or in a recipe with other factors to constrain bacterial wilt disease [13,14].

Extracellular polysaccharides secreted by *R. solanacearum* were used as a specific elicitor of defense responses against bacterial wilt disease of tomato [15]. Many factors play roles in the virulence of *R. solanacearum*, in particular the secretion system effectors (type II and III), N-acetylated extracellular polysaccharide I, and twitching and/or swimming motility (type IV pili-driven twitching and flagella-swimming motility), and cell-wall-degrading enzymes [16].

The bacterial supernatant can be used to manage wilt in potato plants, in this respect, the supernatant of *Saccharomyces cerevisiae* drastically lessened the disease rate of bacterial wilt disease [17]. The virulent strain of *R. solanacearum* may spontaneously convert to an avirulent strain in vitro and in vivo. On the opposite side, the reversal mutation of the avirulent strain into a virulent one has recently been reported [6,18]. On such a basis, the use of supernatant for the avirulent strain of *R. solanacearum* may represent suitable protection against the virulent strain.

The virulent supernatant of microorganisms can be used to control diseases after thermal treatment, unfortunately, the temperature has a huge destroying effect on the cell structure and physiology, however, membranes, proteins, enzymes, DNA, and RNA are also affected [19]. The boiling process of the microbial supernatant led to the breaking of some compounds and/or reactions between different components present in the supernatants [20,21]. The high temperature also led to damage and alteration of the bacterial cell membrane and its components [19]. Therefore, the avirulent strains could be applied as a safe protection agent, without prior treatment.

This study aimed to manage the wilt disease of potatoes, caused by the virulent *R. solanacearum*, using the supernatant and/or cells of an avirulent strain of the same bacterium. According to the authors’ knowledge, there is no preceding trial that uses bacterial supernatant or dead cells of an avirulent *R. solanacearum* in the biocontrol of the virulent strain of bacterial wilt disease, therefore, this study is considered a pioneer in this area.

## 2. Materials and Methods

All chemicals and components of the used media were purchased from Sigma-Aldrich Company (Sigma-Aldrich, Inc., St. Louis, MO, USA).

### 2.1. Bacterial Pathogen

Potato tubers displaying distinctive symptoms of brown rot were washed with sterilized tap water and dried with filter paper, and then surface sterilization was performed using 2% sodium hypochlorite, and rinsing three times with sterile tap water. The bacterial isolation was carried out by transferring a loopful of bacterial ooze from an infected sterilized potato, onto the triphenyl tetrazolium chloride agar medium (TTC) [22], and then incubated for 48 h at 28 ± 2 °C. The grown colony was picked up and re-streaked onto plates of the same medium to obtain pure cultures of the colonies. Bacterial isolate with a white appearance and a red center is regarded as virulent as *R. solanacearum*, which was later identified according to morphological, biochemical, and physiological examinations [23,24]. The isolated pathogen was also molecularly identified.

### 2.2. Molecular Identification

DNA was extracted by centrifugation (8000× *g* for 2 min) of one mL cell suspension, by washing the cells twice with 400 µL of STE Buffer (100 mM NaCl, 10 mM Tris/HCl, 1 mM EDTA, pH 8.0), and centrifuged (8000× *g* for 2 min), and then resuspended in 200 µL TE buffer (10 mM Tris/HCl, 1 mM EDTA, pH 8.0), and 100 µL Tris-saturated phenol (pH 8.0) was added to the tube, and vortexed (60 s) for lysing bacterial cells. Finally, the aqueous phase was separated from the organic one by centrifugation (4 °C, for 5 min at 13,000× *g*). The aqueous upper phase (160 µL) was transferred to a 1.5 mL tube, to which TE buffer (40 µL) was added followed by chloroform (100 µL) and centrifuged (4 °C, for 5 min at 13,000× *g*) [25].

For 16S ribosomal RNA analysis, PCR amplification mixture contained 1× reaction buffer, 1.5 mM MgCl_2_, 1U *Taq* DNA polymerase (Promega), 2.5 mM dNTPs, 30 poml of individual primer, and 30 ng genomic DNA. The primer codes were 27F (5′-AGAGTTTGATCCTGGCTAG-3′), and 1492R (5′-GGTTACCTTGTTACGACTT-3′) with a product size of 1500 bp. The thermo-cycling program was completed in a Perkin-Elmer/GeneAmp^®^ PCR System 9700 (PE Applied Biosystems, Waltham, MA, USA) automated to an initial denaturation cycle (5 min at 94 °C). Followed by 40 cycles, each cycle involved denaturation (30 s at 94 °C), annealing (30 s at 45 °C), and elongation (1 min at 72 °C) steps. The extension segment of the primer stretched to 7 min at 72 °C in the final cycle.

In the detection of the PCR, the amplification outcome was determined by electrophoresis (1.5% agarose gel, including 0.5 µg/mL ethidium bromide in 1X TBE buffer) at 95 volts. A DNA ladder (100 bp) was utilized as a molecular standard. Visualization, and photographing of PCR segments in the UV light as accomplished by Gel Documentation System (BIO-RAD 2000).

The reaction for purification of the amplified PCR was carried out in a 1.5 mL microfuge tube containing the amplified PCR and three volumes of binding buffer 1. The mixture was transferred to the EZ-10 spin column and left (2 min) at room temperature, the column was then washed (750 µL of wash solution), and centrifugated (10,000 rpm for two min), the latter process was repeated to eliminate any residual of wash solution. Then, elution buffer (50 µL) was added and incubated at room temperature (2 min), and the purified DNA was stored (−20 °C).

The PCR sequencing process was performed, following the protocols supplied with the automatic sequencer ABI PRISM 3730XL Analyzer using Big Dye TM Terminator Cycle Sequencing Kits. Rbcl forward primer was applied to perform single-pass sequencing. The ethanol precipitation protocol was applied to purify fluorescent-labeled fragments from the unincorporated terminators and then resuspended in distilled water for electrophoresis (ABI 3730xl sequencer, Microgen Company). The sequence was investigated computationally utilizing the BLAST program (http://www.ncbi.nlm.nih.gov/BLAST (accessed on 14 Jule 2022)), and aligned by Align Sequences Nucleotide BLAST.

### 2.3. Avirulent R. solanacearum

To obtain avirulent strain, the virulent strain was stored on the nutrient glucose agar (NGA) slant and kept for 3 weeks, and then streaked onto TTC plates and checked for the development of the avirulent type that has deep red colonies. Both strains were propagated onto NGA plates and incubated at 28 ± 2 °C for 48 h. For storage of the two strains, a loopful of each strain was transferred to a screw test tube containing sterilized tap water and kept at room temperature [26].

### 2.4. Lytic Activity of Bacterial Strains

#### 2.4.1. Culturing Technique

The two bacterial strains were screened for the production of lytic enzymes in a nutrient glucose broth medium. The flasks were injected with 1.0 mL of bacteria inoculum (10^8^ cfu/mL). The inoculum was prepared from a 2-day-old culture. After incubation (3 days at 30 °C), the supernatant was then separated through centrifugation (5000 rpm for 20 min). The resulting supernatant was examined for lytic activity.

#### 2.4.2. Assay of Hydrolytic Enzymes

Xylanase and filter paperase (FPase) were assayed in the cultural supernatant, xylan, and microcrystalline cellulose, correspondingly, served as substrates. The substrates (0.5%) were solely mixed in 0.05 M citrate buffer (pH 4.8). The supernatant and substrate-buffer solution were mixed (1:1 *v/v*), and incubated for 30 (xylanase), and 60 min (Fpase), at 50 °C. The activity of α-amylase and polygalacturonases (PGase) were detected in the same way, using 1% soluble starch (in citrate phosphate buffer pH 6.6), and 1% pectin (in 0.1 M sodium acetate buffer, pH 5.2), respectively, after incubated for 30 min at 40 °C. The released reducing groups of the four enzymes were determined spectrophotometrically at A_575_ with 3,5-dinitrosalicylic acid method [27,28,29,30]. The unit (U) of the enzyme was expressed as the quantity of enzyme needed to release 1 µmol/g/min of reducing units of xylose (xylanase), glucose (Fpase, and α-amylase), or polygalacturonic acid (PGase) under the assay conditions.

Protease was measured in the bacterial supernatant utilizing casein as a substrate. The liberated amino acids after 10 min incubation at 37 °C were split by trichloroacetic acid and measured at A_280_ [31]. Protease U was described as the quantity of the lipase that liberates 1 µg tyrosine/g/min under the test conditions.

### 2.5. Dual Culture Technique

To discover the presence of any kind of antagonism between the virulent and avirulent *R. solanacearum* strains, the antagonistic activity was conducted [32] with some modifications. The avirulent isolate was inoculated on NGA plates at 3 cm from the plate edge against the virulent isolate of *R. solanacearum*, and then incubated (28 ± 2 °C for three days). The plate was investigated for the presence of an antagonism region between the two bacterial isolates.

### 2.6. Preparation of Treatments

For soil infestation, the virulent strain was inoculated in NGB and incubated (28 ± 2 °C) under shaking (120 rpm, for 48 h). Bacterial cells were separated by centrifugation (10,000× *g* for 20 min) and washed two times with sterilized tap water, and then the bacterial cells were re-suspended in sterilized water, to a final count of 10^8^ cfu mL^−1^ that was used for infestation of the soil in pots.

For the preparation of various treatments, virulent and avirulent isolates of *R. solanacearum* were inoculated in 250 mL NGB in 500 mL flasks. The cultures were incubated (28 ± 2 °C) under shaking (120 rpm, for 48 h). Half of the grown cultures of the two bacteria (virulent and avirulent culture) were boiled in a water bath (100 °C, for 20 min) to kill bacterial cells. The boiled culture was left to cool at room temperature. The boiled and the non-boiled cultures were centrifuged (10,000× *g* for 20 min). The supernatants of both cultures were separated and sterilized using DWK 47 mm filtering apparatus with fritted glass and a 500 mL funnel (United Scientific Supplies, Libertyville, IL, USA) and used in the cell-free extract treatments. The precipitated bacterial cells were washed as described above and resuspended in sterilized water to yield 10^8^ cfu mL^−1^.

### 2.7. Pathogenicity and Virulence Tests on Tomato Plants

Pathogenicity tests and virulence capability tests were performed on tomato seedlings to test the ability of different virulent and avirulent strains to cause the welting symptoms. The tomato pot experiment was applied at TagElezz Research Station, Dakahlia Governorate, Egypt (30°57′25″ N; 31°35′54″ E) in plastic bags (35 cm in diameter) filled with 20 kg of steam sterilized silt clay soil (25% sand: 41.5% silt: 33% clay).

Tomato seedlings (*Solanum lycopersicum* cv. Red gold) at the 3-leaf stage were inoculated with 100 μL each, just above cotyledons using a one ml insulin syringe. Six seedlings were used for each treatment. The various forms of virulent and avirulent strains were control (water), the whole culture, supernatant only, boiled of the whole culture, boiled supernatant only, and dead cells only. The wilting symptoms were observed for three weeks. Wilt symptom progress was recorded daily according to six disease rate classes, i.e., no wilt (0), one leaf wilted (1), two or more leaves wilted (2), all leaves except the tip wilted (3), hole plant wilted (4), and collapse, and death of the plant (5) [33].

### 2.8. Potato Pots Experiment

Tubers of potato stock of cultivar Spunta (Agrico Co. the Netherlands), highly susceptible to bacterial wilt infection, were utilized in this investigation. The soil of the pot experiment was prepared as mentioned above. The experiment was initiated in the 2020 summer season. First, all pots were infested by mixing the upper layer of the soil with 100 mL of a virulent strain of *R. solanacearum* (10^8^ cfu mL^−1^) and regularly irrigated with water for 48 h to facilitate the spreading of *R. solanacearum*. Potato tubers were surface sterilized in 2% sodium hypochlorite and then washed and left to dry. A sharp, clean knife was used to slice potato tubers into pieces of equal size (approx. 2 inches square, for each), containing at least 2 to 3 active eyes. The healthy potato pieces were soaked for 30 min just before planting in different treatments as follows; (1) soil-infection only (control), (2) the whole culture of the avirulent strain (AV), (3) boiling the whole culture of the avirulent strain (BAV), (4) supernatant of the avirulent only (SAV), (5) boiled of avirulent supernatant only (SBAV), (6) dead avirulent cells only (DCAV), (7) virulent supernatant only (SV), (8) boiled of the whole virulent culture (BV), (9) boiled of virulent supernatant only (SBV), and (10) dead virulent cells only (DCV). Two potato tuber pieces were planted deep-on in each bag and grown in a greenhouse (100 days at 22 °C and 34 °C). A completely randomized block design was applied.

#### 2.8.1. Physiological Performance

Total phenol was determined after 40 days from planting with Folin–Ciocalteu reagent [34]. A fresh weight of 0.05 g of shoot tissue was ground in 10-times the volume of ethanol (80%) using and mortar, and pestle then centrifuged (for 20 min at 10,000 rpm). The filtrate was kept and the residue was treated again with 5-times of ethanol (80%) and centrifuged. The filtrates were collected and evaporated to dryness. The deposit was liquified in distilled water (5 mL). The total phenol was detected in a reaction mixture composed of 0.2 mL plant filtrate, 2.8 mL of distilled, and 0.5 mL of Folin reagent. After 3 min, Na_2_CO_3_ solution (20%) was also mixed thoroughly with the contents, and then boiled (one min in a water bath), and cooled. The absorbance was detected at A_650_ nm [35].

#### 2.8.2. Assay of Defense Enzymes

The defense-related enzymes were extracted from plant leaves in Tris-HCl buffer (0.05 mM, pH 8.4) containing 15 mM β-mercaptoethanol, using a homogenizer, followed by centrifugation (10,000 rpm for 20 min) at 4 °C [36]. The supernatant was used for the estimation of enzymes.

The reaction mixture of polyphenol oxidase (PPO) is composed of plant extract, 0.2 M potassium phosphate buffer (pH 7), and catechol as a substrate (1:2:1, *v/v*). The change in absorbance was spectrophotometrically measured at A_420_ nm. The unit of the enzyme activity (U) was stated as ΔA_420_ min/g fresh weight under the assay conditions [37].

The assay mixture of peroxidase activity (POD) consisted of 0.5 mL of plant extract, 1 mL of phosphate buffer (pH 7.1), 0.5 mL of pyrogallol, and 0.5 mM of H_2_O_2_. The developed color as a result of the pyrogallol oxidation was measured spectrophotometrically. The unit of the enzyme activity (U) was stated as ΔA_470_ min/g fresh weight under the assay conditions [37].

#### 2.8.3. Disease Rating

The symptoms of wilt severity on potato plants were rated daily and recorded based on the following scale; 0 = no symptoms, 1 = up to 25% wilt, 2 ≤ 25 to 50% wilt, 3 ≤ 50 to 75% wilt, and 4 ≤ 75 to 100% of the foliage wilted [38].

#### 2.8.4. Growth, and Yield

After 70 days from planting, the height, number of branches, and dry weight of shoot (60 °C till constant weight). At harvesting time, the number and weight of tubers were estimated.

The leaf area index (LAI) measurement was performed using the disk method. The total fresh weight of plant leaves was determined. The whole leaf area was estimated with known desk diameter, discarding all fractional disks, thus the leaf area/weight relationship was calculated [39].

Latent tuber infection was examined for the resulting potato tubers from various pot experiments after storage (6 weeks) at room temperature to allow the symptom development, and facilitate the detection of latent tuber infection [40]. Then, the tubers were cut to estimate the number of infected tubers, and the percentage of latent tuber infection was calculated.

### 2.9. Gas Chromatography–Mass Spectrometry (GC–MS)

Four treatments, SV, SAV, BSV, and BSAV were analyzed via GC–MS, using Agilent 6890 gas chromatograph (Agilent, Santa Clara, CA, USA) equipment with an Agilent mass spectrometric detector, with a direct capillary interface and fused silica capillary column PAS-5MS (30 m × 0.32 mm × 0.25 μm film thickness). Carrier gas (helium) was at 1 mL/min, pulsed split less mode. The solvent delay and the size of the injection were 3 min and 1 μL, respectively. The mass detector of the spectrophotometry was performed in electron ionization mode of 70 eV voltage over the range of 50 to 500 *m*/*z*. The temperature of the ion source and quadrupole1 were 230 °C and 150 °C, respectively, with an EM voltage of 1650 V. The programmed temperature was initiated at 90 °C (3 min) and then raised at 8 °C/min up to 280 °C. The temperature of the injector and detector were 250 and 280 °C, correspondingly. The mass spectral patterns of the compounds were recognized with WILEY/NIST mass spectral database.

### 2.10. Statistical Analysis

The treatments were performed in six replicates (each replica consists of 3 bags), applying the completely randomized block design. The data were statistically analyzed by one-way analysis of variance, followed by means comparison using Tukey’s HSD test (probability, *p* ≤ 0.05) with the aid of CoStat software (version 6.450, CoHort Software, USA).

## 3. Results

### 3.1. Isolation and Identification

**Figure 1:**
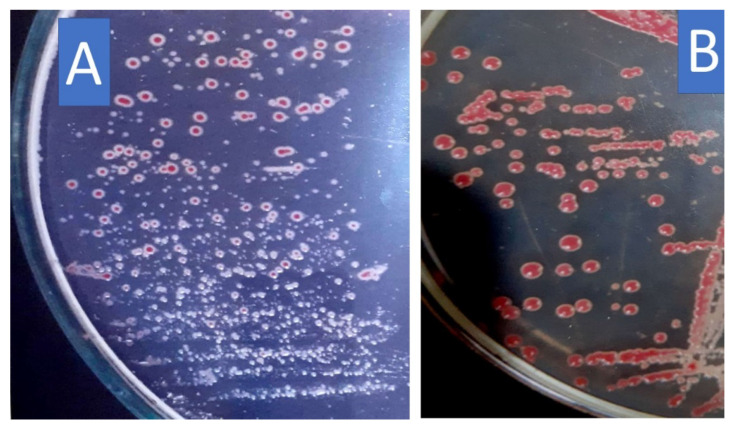
The virulent and avirulent strains of *R. solanacearum* on triphenyl tetrazolium medium, (**A**) virulent colonies appear as highly white-fluidal with a less pink center, or totally white due to the existence of exopolysaccharides, (**B**) avirulent colonies have deep red color and appear to flatten are non-fluidal and have no white exopolysaccharides.

The morphology of the colonies of the virulent strain of *R. solanacearum* is irregular, fluidal, and white with a pink color in the center, or totally white on TTC agar medium while the colonies of the avirulent strain on the same medium are deep red in color and appear to flatten and are non-fluidal. The avirulent strain was obtained through storage of the virulent strain on NGA slant for 3 weeks, the development of an avirulent type that has deep red colonies. Figure 1 shows the differentiation between the colonies of the virulent and avirulent strains of *Ralstonia solanacearum*. Morphological and biochemical characteristics signified that the bacterial isolate had Gram-negative rods, was without fluorescent pigment on King’s B medium, produced H_2_S from cysteine, had reduced nitrate, and gave a positive reaction with catalase and oxidase tests. All isolates were unable to hydrolyze starch and liquefy gelatin. All isolates formed a slight indole formation. The isolates created acid from glucose, cellobiose, fructose, lactose, and maltose but not from D-arabinose, mannitol, salicin, and sorbitol. Accordingly, the isolate was *R. solanacearum* (race 3 biovar 2).

### 3.2. Molecular Identification

**Figure 2:**
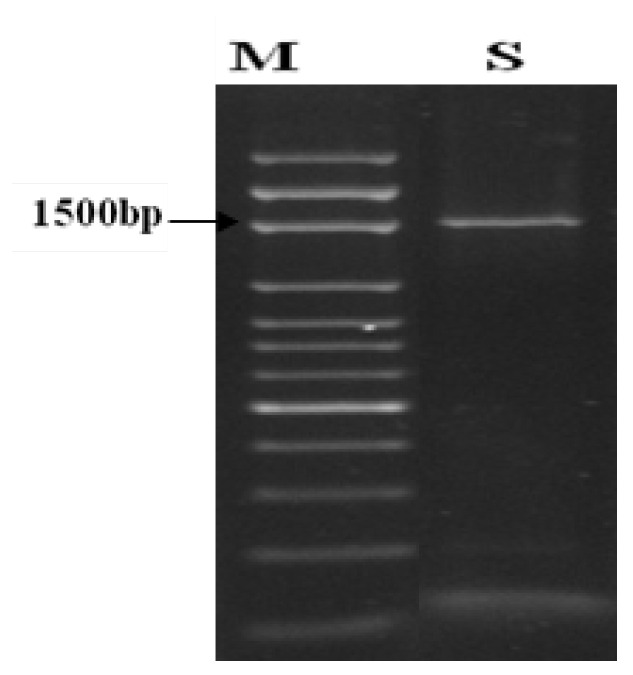
Electrophoresis of the amplified product of PCR of 16s rRNA fragment of the bacterial isolate on the agarose gel. M; marker, and S; sample.

**Figure 3:**
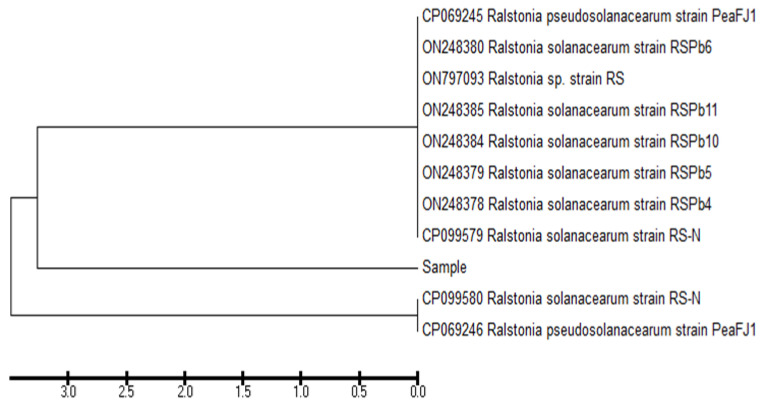
Phylogenetic tree of *R. solanacearum* (accession number; OP180100), using 16S rRNA, showing names of related bacterial species.

The pathogenic bacterial isolate was identified molecularly. After amplification, the PCR product was detected through electrophoresis (Figure 2). The sequence coding for the 16S rRNA gene was assessed. From the Blast assessment, the isolate ZWtag exhibited a high similarity with *R. solanacearum* on the GenBank (Figure 3). Accordingly, the strain was classified as *R. solanacearum* with GenBank accession number OP180100.

### 3.3. Lytic Enzymes Profile

**Figure 4:**
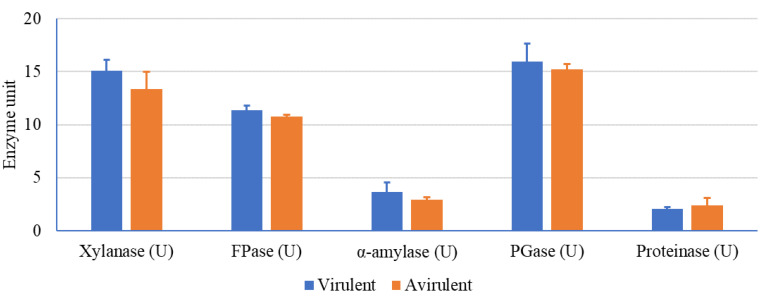
The activity of the hydrolytic enzymes of the virulent and avirulent strains of *R. solanacearum*.

The lytic activity in the cultural supernatant of the virulent and avirulent *R. solanacearum* strains was explored (Figure 4). There was an obvious activity of various tested enzymes of the virulent *R. solanacearum*, being 15.061, 11.397, 3.650, 15.921, and 2.1 U for xylanase, FPase, amylase, PGase, and proteinase, respectively. Additionally, the lytic activity of the avirulent *R. solanacearum* showed activity of 13.354, 10.791, 2.950, 15.247, and 2.4 U for xylanase, FPase, amylase, PGase, and proteinase, respectively. Generally, the virulent strain showed higher lytic activity in most of the tested enzymes, however, proteinase was the only exception.

### 3.4. Pathogenicity of R. solanacearum

For the determination of the virulence ability of both *R. solanacearum* strains to generate the welting symptoms, the pathogenicity test was performed on tomato seedlings as a plant model. As indicated in Table 1, opposite to the control, all treatments led to the development of wilting symptoms in the tomato seedlings. The disease rate varied from one treatment to another. The highest disease rate was recorded with the whole culture being 4.8, however, all the treatments caused wilting, and the lowest value among the treatments was the boiled supernatant only, which recorded a disease rate of three. On the other side, all avirulent treatments failed to develop any disease symptoms in the tomato seedlings.

### 3.5. Dual Test Cultures

Before the greenhouse study, the dual culture test was carried out using the virulent and avirulent strains. The antagonistic behavior of the avirulent isolate against the virulent isolate of *R. solanacearum* is negative where there was no clear zone around the avirulent strain of *R. solanacearum*.

### 3.6. Greenhouse Study on the Potato

#### 3.6.1. Physiological Performance

The physiological performance of potato plants was measured under various treatments. The data in Table 2 indicate that all treatments led to an obvious increase in total phenols, PPO, and POD in plant leaves. Some of these increases are significant and others are non-significant. The highest value of total phenols was recorded with the BV treatment at 56.5 mg/g fresh weight. Then, there was no significant difference among the SBV, SV, and SBAV treatments, which recorded values of 49.88, 42.54, and 41.87 mg/g, respectively. Additionally, there was no significant difference among the control, AV, BAV, SAV, and DCV, which recorded 27.84, 29.18, 37.20, 28.51, and 32.52 mg/g, respectively.

Regarding the defense-related enzymes, the most significant increase in PPO was recorded with SBV (205.33 U) and BV (203.5 U). Oppositely, SV led to the lowest significant activity of polyphenol oxidase (97.78 U), while SAV, SBAV, and DCV had an insignificant activity in polyphenol oxidase. The most significant activity of POD was shown with SBAV, which recorded 30.67 U. The other treatments showed insignificant activities. As a general trend, boiling the whole culture and supernatant of virulent and avirulent strains had values more than those of non-boiling treatments of total phenols, PPO, and POD.

#### 3.6.2. Plant Growth and Wilt Development

For further elucidation of the tested treatments, the growth performance of the potato plants and wilt development were measured after 70 days of planting. The results in Table 3 reveal that AV treatment significantly increased plant height (37.4 cm) with a percentage of increase of 20.65% compared to the control (31 cm), while the other avirulent treatments led to non-significant increases in plant height, being 4.52% (BAV), 2.58% (SAV), 5.16% (SBVA), and 7.10% (DCAV). On the other hand, SV, BV, SBV, and DCV caused a significant reduction in plant height, being 19.35%, 10.97%, 14.19%, and 7.10%, respectively, compared with soil infection only (control). Furthermore, there were no significant variances among treatments in branches number.

As the main purpose of the current work, the disease was rated to assess the different treatments (Table 3). All the avirulent remedies led to a significant reduction in disease rating where the AV treatment is the best in wilt disease management, recording a disease rating of 1.2, followed by SAV and SBAV without significant differences among them. In the contrast, BAV showed no significant variances compared with the control. All applications of the virulent wilt strain did not have any significant effect in reducing wilt disease compared to the soil-infection only (control), and also with most of the avirulent treatments.

The highest significant values of LAI were recorded by AV, SBAV, DCAV, SBV, and DCV treatments, being 0.474, 0.466, 0.470, 0.484, and 0.468, respectively, followed by BAV (0.446). Further, compared with the infected control, significant increments were also observed in plant dry weight by all avirulent strain treatments as well as both SBV and DCV treatments.

#### 3.6.3. Potato Yield and Latent Infection

Regarding the influence of various vaccination treatments on potato yield and tubers’ infections after storage, the data in Table 4 indicate that the number of tubers per plant had no significant variation between all treatments, except only for the DCV treatment being 2.8 tubers per plant. Concerning tuber yield, AV treatment caused a significant increase in the weight of potato tubers (123 g) with an increased percentage of 48.2% than the control treatment (83.4 g). However, such treatment was superior to the other treatments. Next, BAV, SBAV, and DCA led to a lower significant increase in the weight of potato tubers by 11.51, 11.03, and 12.71% compared to the control, respectively. Oppositely, all the virulent strain treatments led to a significant reduction in the weight of tubers where, SV, BV, SBV, and DCV were 38.61, 26.33, 34.53, and 31.18% lower than the control, respectively.

Potato tubers retrieved from the various treatment pots investigated were stored for 6 weeks to follow up on the influence of various treatments on the development of latent infection (Table 4). The percentage of latent infection of stored tubers was significantly reduced by the avirulent treatments, which recorded 54, 62, 73.6, 70.33, 74.33% with decreasing percentages of 42, 34, 22.4, 25.67, and 21.67% for AV, BAV, SAV, SBAV, and DCAV, respectively, compared with the control. In contrast, the latent infection in potato tubers recorded non-significant values with virulent treatments.

### 3.7. GC–MS Analysis

**Figure 5:**
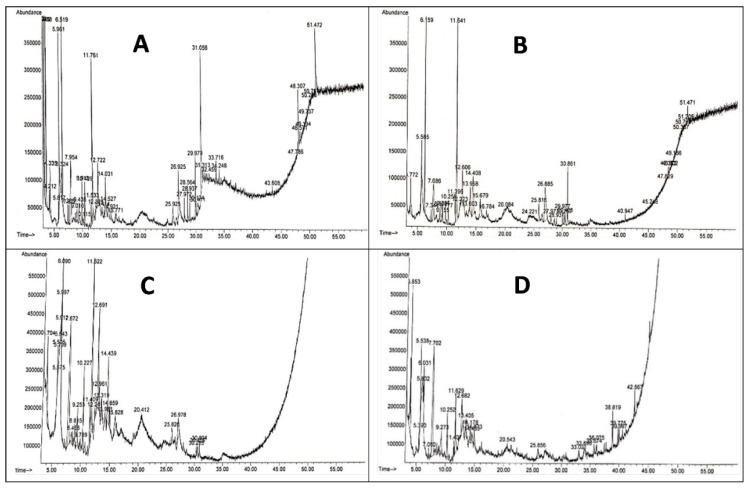
GC–MS chromatogram of bioactive metabolites of the four kinds of bacterial supernatants. (**A**): supernatant of virulent strain, (**B**): supernatant of avirulent strain, (**C**): boiled supernatant of virulent strain, and (**D**): boiled supernatant of avirulent strain.

The analysis of cell-free culture supernatant of the virulent strain led to the identification of 19 compounds (Table 5 and Figure 5A). The main constituents detected were n-hexadecanoic acid (palmitic acid, 10.68%), acetic acid (9.10%), and 4H-pyran-4-one, 2,3-dihydro-3,5-dihydroxy-6-methyl- (8.90%). 2-cyclopenten-1-one, 2-hydroxy- (6.92%), 2-propanone, 1-hydroxy- (Hydroxyacetone) (6.89%), and 2-hydroxy-gamma-butyrolactone (6.77%) were also identified in the virulent strain. Only 11 compounds were detected in the cell-free culture supernatant of the avirulent strain of *R. solanacearum* including 1,3-dioxolane, 2,4,5-trimethyl- (18.03%), 2(3H)-furanone, 5-methyl- (11.30%), 1,2,3-propanetriol, 1-acetate (7.32%), and butanal, 2-methyl- (6.12%) (Table 6 and Figure 5B). The components identified in the boiled supernatant of the virulent strain of *R. solanacearum* were 20 different compounds (Table 7 and Figure 5C). The main compounds detected were 2-hydroxy-gamma-butyrolactone (21.17%), 4H-pyran-4-one, 2,3-dihydro-3,5-dihydroxy-6-methyl (16.59%), and 1,2,3-propanetriol, 1-acetate (7.18%). 2-hydroxy-gamma-butyrolactone (27.78%). The main constituents in the boiled supernatant of the avirulent strain were 2,3-butanediol (15.22%) and 4H-pyran-4-one, 2,3-dihydro-3,5-dihydroxy-6-methyl- (8.83%) (Table 8 and Figure 5D).

## 4. Discussion

Isolation trials identified *R. solanacearum*, which is a worldwide soil-borne pathogen that generates bacterial wilt disease, thus infecting a wide range of plants. Conventionally, *R. solanacearum* could be distinguished by ooze-out, exudation, or bacterial streaming from infested parts of the plant. This bacterium has two phenotypes: virulent and avirulent. The virulence factor of this bacterium can be lost during storage and subculturing, i.e., phenotypic conversion from virulent to avirulent phenotype [6]. Recently, the reversal of the phenotypic conversion from avirulent to virulent was also reported [18].

The molecular identification was carried out with 16S rRNA. All bacteria contain a 16S rRNA gene of about 1500 bp. The gene contains sections of DNA sequence that are exclusive to specific species. The species of an unknown bacterium may be deduced from its unique sequence of rRNA genes. The pathogenic isolate was identified as *R. solanacearum*, and the 16S rRNA sequencing was registered on the GenBank under the accession number OP180100. Molecular identification is usually performed because of its sensitivity and specificity for the rapid identification of various organisms [41]. The sequence used for constructing the phylogeny is totally interpreted and firmly correlated with the other similar bacterial strains in the GenBank database.

Generally, the virulent and avirulent *R. solanacearum* exhibited lytic activity. The virulent strain has a higher lytic activity than the avirulent one. However, the lytic activity of the phytopathogenic microorganisms was found to be linked to pathogenicity thus playing a fundamental task in disease progression, and further, could be utilized as a differentiating tool [31].

Cellulose, starch, pectin, and protein are the main components of plant tissue. Such a structure restricts the invasion of phytopathogens. That is why lytic enzymes play an essential role in the pathogenesis process. Therefore, xylanase, FPase, amylase, and proteinase were measured since these enzymes are known as cell wall-degrading enzymes and are involved in the pathogenicity process to facilitate microbial penetration into plant tissue [29,42]. The great disparity in the enzymatic profile among the virulence and virulence strains may be due to genetic variation and ecological conditions.

Cellulose is degraded by the lytic activity of cellulase into a single monomer of glucose [29,31]. Pectin is decomposed of galacturonic acid monomers and catalyzed by PGase by cleaving the α-1,4-glycosidic bond [30,31]. Starch is decomposed by amylase, leading to the liberation of glucose units [42]. Finally, the nitrogenous part of the plant is hydrolyzed by proteases into peptides and amino acids [42]. The synergistic action of the enzymatic consortium causes the maceration of plant tissue, thus facilitating the infection by the pathogen, and therefore, may be essential for pathogenicity. Lytic enzymes represent an additional threat to plant infection. In summing up, the current pathogen has a complementary profile of lytic enzymes, capable of causing a serious infection.

Some studies investigated the use of the avirulent strain of fungal plant pathogens to control the corresponding diseases such as the use of the avirulent *Colletotrichum* to induce systemic resistance in strawberries [43]. Similarly, different investigations indicated that non-pathogenic Fusarium can be used for the biological control of many diseases that have developed by the pathogenic strains, the mode of action of this biocontrol may be antibiosis, plant growth promotion, and/or induced systemic resistance. Secondary metabolites of such fungi can be used for biological control [44]. Additionally, the avirulent, *Fusarium oxysporum* f. sp. *lycopersici*, can reduce susceptibility to virulent strains by activating either I, I-2, or both resistance proteins upon recognition of Avr1 of the Avr2/Six5 pair, respectively [45]. The current investigation reported the first use of a bacterial pathogen as an inducer of plant systemic resistance.

The current study reported negative antagonism between the virulent and the virulent *R. solanacearum*, since the avirulent strain of *R. solanacearum* did not antagonize the virulent strain and it does not perform bacteriocinogenic action [46]. Therefore, the avirulent isolate of this study might not be bacteriocinogenic. Contrasting results found that the avirulent strain antagonized the virulent strain of *R. solanacearum* [10,47]. Accordingly, the proposed mode of action of the current biocontrol activity is restricted to the induced systemic resistance of plants by the avirulent *R. solanacearum*.

The virulence ability is generally or partially attributed to the presence of fatty acids. Besides playing an important role in intercellular communication signals, fatty acids play a vital role in motility, which is important in the virulence system. The linkage and arrangement of fatty acids to the membrane propose a mechanism of swarming through the control of membrane fluidity [48]. For instance, palmitic acid and its derivatives were found and play a central task in the virulence of *R. solanacearum* [49,50].

The main constituent of the avirulent *R. solanacearum* is “2(3H)-Furanone, 5-methyl”, which was reported to play a vital role in antimicrobial activity against *R. solanacearum* and numerous other plant pathogenic bacteria [51]. Although there was no antagonism between the current virulent and avirulent *R. solanacearum*, there is another advantage of using the avirulent strain in the management of other microbial pathogens through its butanal, 2-methyl, which was reported as a valuable constituent in the biological control of plant disease [52].

The pathogenicity test and welting capability of *R. solanacearum* on tomato seedlings revealed that all the used forms of the virulent strain led to the spread of the wilting disease. The highest recorded disease rate was observed with the whole culture of the virulent strain, while only the boiled supernatant led to the lowest value of disease rate. All treatments showed an ability to affect the wilting disease, presumably due to the presence of several components of virulence factors, what is more, the virulence factors of the pathogenic bacterium are distributed between the bacterial cell and its secreted supernatant, consequently, the presence of both are complementary and required for the complete development of the welting symptoms and disease as well. According to our knowledge, the current work is the first comprehensive study that elucidates the occurrence of the virulence factor in *R. solanacearum* by using the virulent strain and/or its supernatant to test their pathogenic activities. Oppositely, the avirulent strain did not develop any disease symptoms in either the cell or the supernatant, suggesting that the avirulent strain could be used safely for the biological administration of brown rot disease caused by the virulent strain.

The application of both forms of *R. solanacearum* on potatoes shows that the physiological features of the plants in terms of total phenols and the defense-related enzymes (PPO, and POD) increased with all treatments, especially those of virulent treatments than the corresponding avirulent treatments. These results suggest that induced resistance was highly simulated in the plants infected with the virulent strain, and this may be due to the increment of plant defense with the increase in the threat of pathogens. Phenolics are secondary secretions with various roles in the mitigation of many abiotic and biotic stresses. Phenolic compounds have an antimicrobial action in plants, e.g., safeguarding against disease causatives such as microbes, weeds, nematodes, and others [53,54,55]. Therefore, the infection by virulent *R. solanacearum* and/or supernatant (boiled or non-boiled) led to an increase in biotic stress that led to increases in plant phenolic compound production as a response to such stress, compared with the avirulent strain. Furthermore, the phenolic compound may display antimicrobial and antioxidant activity that helps to evade pathogenic infections and induce resistance to protect the plant tissues from the toxic effect of reactive free radicals [56,57].

PPO and POD are defense-related enzymes, acting as biocontrol agents by increasing the immune response of the plant. This finding has previously been confirmed in three bacterial species (*Bacillus subtilis*, *B. polymyxa*, and *Pseudomonas fluorescens*), which did not inhibit the growth of *R. solanacerum* but induced systemic resistance by increasing phenolic compounds, peroxidase, PPO and POD [58]. Another investigation stated that the phenolic compounds, peroxidase, PPO, and POD increased when bacterial wilt of potato was managed by the biocontrol agent Saccharomyces cerevisiae (cells and supernatant) without antagonizing *R. solanacerum* [17]. Therefore, the proposed application of the avirulent *R. solanacearum* may biologically act as an inducer of plant defense.

The significant alteration occurred in potato growth due to the application of different virulent and virulent *R. solanacearum*, of which virulent treatments significantly decreased the plant growth. Treatment with the avirulent strain of *R. solanacearum* had a significant increase in plant height than other virulent treatments and the control. Similarly, the vaccination of the tomato plant leads to an increase in plant height by 13.71% [14]. However, boiling and/or filtration such as in the case of BAV, SAV and SBAV led to an insignificant increase in plant height; this may be due to the reduction of the pathogenicity effectiveness of AV strain as a result of boiling and filtration. The number of branches showed insignificant alteration. As a response to potato growth promotion, a positive significant effect of avirulent and DCV treatments was recorded on LAI. The LAI is a significant sign of precipitation, radiation interception, energy conversion, and balance of water. Eventually, it is a reliable issue for plant growth and one of the main driving forces of nutrient, water usage, and carbon balance, consequently, on net production [59]. Therefore, there was an obvious internment plant dry weight with the increments on LAI.

Pathologically, the virulent treatments increased the disease symptoms on potato plants compared to the avirulent treatments. This is an expected result since the pathogen and/or its secretions contain virulence factors such as components that cause pathogenicity and can suppress the plant defenses. Pathogens create proteins, called effectors, which go into the plant tissue to lower the defense response of plants, which enables the pathogen to succeed in infecting the host. These effectors can inhibit several metabolic or signaling pathways of the host response thus facilitating pathogen infection [60]. Additionally, *R. solanacearum* can metabolize ferulic acid and salicylic acid to protect itself from the toxic action of phenolic acids [61]. Another protective mechanism of the metabolites and cells of the bioagents suggests the creation of reactive phytoalexins, phenolic compounds, and/or the formation of physical barriers such as adjustments of cell walls and cuticles or pathogenesis-related proteins by the induced plant [62].

The significant decrease in disease caused by SAV treatment may be due to the SAV-containing components that play a role in the inhibition of virulent *R. solanacearum*. While the insignificant decrease in wilt disease caused by BAV treatment may be due to the alteration of components of BAV by heat and a possible reaction between these components since the high temperature led to destroying the membrane and changing the chemical composition of the heat-treated treatments [19].

The SV treatment had the highest value of wilt disease rate, which may be due to the presence of components that can cause disease and increase the symptoms of bacterial wilt disease and increase the dwarfing of the potato plants. The boiling, as aforementioned, can change the components of the supernatant and decrease the pathogenic effect of the SV treatment, therefore, the BV and SBV had less pathogenic effect than the SV. Additionally, DCV may have a pathogenic effect on the potato plant but less than the supernatant (boiled and non-boiled).

At the end of the experiment, the avirulent treatments significantly surpassed other treatments in the productivity of potatoes and significantly decreased the infected tubers with brown rot. Plant tissues with high intensity of avirulent *R. solanacearum* banned or hindered the entry of the virulent pathogen since the avirulent strain of *R. solanacearum* can invade and enter the plant host and occupy all space without injury to the root, hindering and delaying the multiplication of the virulent strain of *R. solanacearum* [9,12]. Furthermore, the presence of avirulent *R. solanacearum* increases the availability of soil nutrients by changing the soil conditions into neutral; the latter is one of the main factors for the management of bacterial wilt disease [13]. These effects may be due to phytohormones and other substances secreted by the avirulent strain of *R. solanacearum* that act like bioagents in encouraging plant growth and productivity, as well as decreasing bacterial wilt disease [17,58].

The increase in brown rot disease of potato tubers and decrease in potato tubers productivity in virulent treatments may be due to the increase in exopolysaccharides that are the major virulence factor, causing wilting of the host plant due to plugging the plant vessels [6] and an increase in enzymes and other components secreted by the virulent *R. solanacearum* [16,60,63]. Our results reveal no significant alteration in the tubers’ number at harvesting; however, there was some decay in the tubers, which reflected on the weight only, but none of them were totally decayed, therefore, there was no significant change in the tubers’ number. In contrast, a significant decrease in the percentage of latent infection of stored tubers was recorded by avirulent bacterial treatments.

The results of GC–MS analysis demonstrated that the fatty acids or their derivatives (fatty acid methyl esters and fatty acid ethyl esters) were detected in all types of cell-free culture supernatant of the virulent and avirulent strains of *R. solanacearum*. Fatty acids are vital biomolecules that play a central role in almost all organisms and act as signaling means in plant–microbe interactions and are involved in important physiological functions in cells. For instance, several studies have revealed that palmitic acid, a saturated long-chain fatty acid compound, has shown remarkable antibacterial properties towards Gram-positive and Gram-negative bacteria [64] as well as inhibition of the growth of the plant pathogenic fungi and promoting the growth of seedlings [65,66]. The mechanisms of antimicrobial action by palmitic acid are related to increasing cellular toxicity and the rate of cell death with reduced ergosterol content in the plasma membrane by inducing reactive oxygen species (ROS). The building up of ROS causes severe damage to cellular DNA, RNA, and protein levels as well as alters the virulence processes of the cell [67].

The compound 4H-Pyran-4-one, 2,3-dihydro-3,5-dihydroxy-6-methyl- = 2,3-dihydro-3,5-dihydroxy-6-methyl-4H-pyran-4-one (DDMP) was found in all cell-free supernatants except the avirulent strain supernatant. This compound is one of the primary bitter compounds that was identified to be generated in the intermediate stage of the Maillard reaction and its formation is related to sugars (fructose, and glucose), amino acids (tyrosine, proline, histidine, and lysine) [68]. The toxicological relevance of DDMP is not clear, but it is thought to generate free radicals that damage DNA through self-oxidation, another DDMP that has a strong antioxidant effect and other activities [69].

In general, the antimicrobial activity of the avirulent supernatant may be owing to the major constituents or the collaborative effects between the minor and major components [70]. The efficacy of the combination exceeds the predictable cumulative efficacy of the separate component alone.

It is noticeable that the boiling process results in a difference in the activity and composition of the supernatant of both the virulent and avirulent strains of *R. solanacearum*. It is possible that the boiling process leads to the breaking of some compounds and/or a reaction between different components present in the bacterial supernatant, or may cause damage and breakdown of extracellular polysaccharides of the virulent strain, leading to the loss of virulence. Furthermore, the boiling process enhances the total phenolic content, and antioxidant activities due to the liberation of bound phenolic substances and the creation of intermediate generated owing to the Maillard reaction products and decreased the content of individual functional components owing to their thermal decomposition. Another reason is the elevated sugar content and reduced total free phenols and fatty acids [71]. All these changes in components in the boiled supernatants led to a change in their effects on the plant in this investigation.

It is worth mentioning the reversal possibility of the avirulent strain to the virulent strain type [6,18]. That possible reversion represents a limitation for the wide application of the avirulent strain. Our paper, therefore, suggests an innovative simple application through using thermal treatment (boiling) or cell-free supernatant to simply overcome this possible reversion problem and gave alternative forms of the avirulent *R. solanacearum* to manage the bacterial wilt of potatoes and other plants. What is more, this method can be generalized to manage bacterial diseases in plants, animals, and human beings, which may be useful tools for biological control.

## Figures and Tables

**Table 1:** Virulence capability and disease rating of the various forms of the virulent and avirulent strains of *R. solanacearum* on tomato seedlings.

Treatment	Disease Rating Caused by *R. solanacearum*	
	Virulent	Avirulent
Uninfected control (Water)	0.0	0.0
Whole culture	4.8 ± 0.45	0.0
Boiled of the whole culture	3.8 ± 0.84	0.0
Supernatant only	4.6 ± 0.55	0.0
Boiled supernatant only	3.0 ± 0.71	0.0
Dead cells only	3.2 ± 1.30	0.0

**Table 2:** Physiological characteristics (per gram fresh weight) of potato leaves after 40 days from planting.

Treatment	Total Phenols (mg/100 g)	Polyphenol Oxidase (U)	Peroxidase (U)
Soil infection (control)	27.84 d	5.33 d	22.67 ab
AV	29.18 d	171.56 b	10.67 b
BAV	37.20 cd	164.45 b	14.67 ab
SAV	28.51 d	10.66 d	9.33 b
SBAV	41.87 bc	20.44 d	30.67 a
DCAV	37.19 cd	176.00 ab	10.67 b
SV	42.54 bc	97.78 c	22.67 ab
BV	56.50 6a	203.56 a	11.33 b
SBV	49.88 b	205.33 a	4.67 b
DCV	32.52 d	20.44 d	3.33 b

The whole culture of the avirulent strain (AV), the boiled whole culture of the avirulent strain (BAV), supernatant of the avirulent only (SAV), boiled of avirulent supernatant only (SBAV), dead avirulent cells only (DCAV), virulent supernatant only (SV), boiling of the whole virulent culture (BV), boiled of virulent supernatant only (SBV), dead virulent cells only (DCV). Different letter(s) in the same column indicates significant variances based on Tukey’s test at *p* ≤ 0.05.

**Table 3:** Growth parameters and disease rating after 70 days of potato planting.

Treatment	Plant Height (cm)	Branch No. Per Plant	Leaf Area Index	Dry Weight (g/Plant)	Disease Rating
Soil infection (control)	31.0 bc	1.20 a	0.394 d	26.50 cd	3.8 a
AV	37.4 a	2.20 a	0.474 a	30.00 a	1.2 e
BAV	32.4 b	2.50 a	0.446 bc	28.60 ab	3.6 a
SAV	31.8 bc	2.50 a	0.428 c	28.20 ab	1.8 de
SBAV	32.6 b	1.60 a	0.466 ab	29.20 a	2.2 cde
DCAV	33.2 b	2.50 a	0.470 ab	28.40 ab	2.4 bcd
SV	25.0 e	1.20 a	0.378 d	24.50 d	4.0 a
BV	27.6 de	1.20 a	0.382 d	26.6 bc	3.8 a
SBV	26.6 de	1.40 a	0.484 a	30.0 a	3.4 ab
DCV	28.8 de	1.80 a	0.468 ab	28.60 ab	3.2 abc

The whole culture of the avirulent strain (AV), the boiled whole culture of the avirulent strain (BAV), supernatant of the avirulent only (SAV), boiled of avirulent supernatant only (SBAV), dead avirulent cells only (DCAV), virulent supernatant only (SV), boiling of the whole virulent culture (BV), boiled of virulent supernatant only (SBV), dead virulent cells only (DCV). Different letter(s) in the same column indicates significant variances based on Tukey’s test at *p* ≤ 0.05.

**Table 4:** Number and weight of tubers at harvesting, as well as latent infection percentage of potato tuber after 6 weeks of storage.

Treatment	Tubers’ Number/Plant	Weight of Tubers (g/Plant)	Latent Infected Tubers %
Soil infection (control)	4.4 ab	83.4 c	96.0 a
AV	4.6 ab	123.6 a	54.0 e
BAV	5.8 a	93.0 b	62.0 de
SAV	4.4 ab	92.0 bc	73.6 cd
SBAV	3.4 ab	92.6 b	70.3 de
DCAV	3.6 ab	94.0 b	74.3 cd
SV	3.8 ab	51.2 e	100.0 a
BV	4.2 ab	61.4 d	92.0 ab
SBV	3.0 ab	54.6 de	91.0 abc
DCV	2.8 b	57.4 de	95.0 a

The whole culture of the avirulent strain (AV), the boiled whole culture of the avirulent strain (BAV), supernatant of the avirulent only (SAV), boiled of avirulent supernatant only (SBAV), dead avirulent cells only (DCAV), virulent supernatant only (SV), boiling of the whole virulent culture (BV), boiled of virulent supernatant only (SBV), dead virulent cells only (DCV). Different letter(s) in the same column indicates significant variances based on Tukey’s test at *p* ≤ 0.05.

**Table 5:** GC–MS analysis of bacterial supernatant of the virulent strain of *Ralstonia solanacearum*.

No.	Rt ^a^	Compound ^b^	Molecular Formula	Molecular Weight	Peak Area (%)
1	3.24	Methyl glyoxal	C_3_H_4_O_2_	72.06	4.33
2	3.41	Acetic acid	C_2_H_4_O_2_	60.05	9.10
3	3.66	2-Propanone, 1-hydroxy- (Hydroxyacetone)	C_3_H_6_O_2_	74.07	6.89
4	4.33	4-Penten-2-one, 4-methyl-	C_6_H_10_O	98.14	2.10
5	6.51	2-Cyclopenten-1-one, 2-hydroxy-	C_5_H_6_O_2_	98.09	6.92
6	7.95	2-Hydroxy-gamma-butyrolactone	C_4_H_6_O_3_	102.08	6.77
7	9.01	1,3-Dioxol-2-one,4,5-dimethyl-	C_5_H_6_O_3_	114.09	0.52
8	9.43	Furaneol	C_6_H_8_O_3_	128.12	1.45
9	11.76	4H-Pyran-4-one, 2,3-dihydro-3,5-dihydroxy-6-methyl-	C_6_H_8_O_4_	144.12	8.90
10	14.03	5-Hydroxymethylfurfural	C_6_H_6_O_3_	126.11	3.21
11	14.53	1,2,3-Propanetriol, 1-acetate	C_5_H_10_O_4_	134.13	1.71
12	26.92	Tetradecanoic acid (Myristic acid)	C_14_H_28_O_2_	228.37	3.11
13	27.96	9-Undecen-2-one, 6,10-dimethyl-	C_13_H_24_O	196.32	0.16
14	28.56	2-Undecanone, 6,10-dimethyl-	C_13_H_26_O	198.34	1.29
15	28.93	Undecanal	C_11_H_22_O	170.29	0.70
16	29.98	Hexadecane	C_16_H_34_	226.44	2.13
17	30.17	Hexadecanoic acid, methyl ester	C_17_H_34_O_2_	270.45	0.40
18	31.05	n-Hexadecanoic acid (Palmitic acid)	C_16_H_32_O_2_	256.42	10.68
19	33.71	1-Octadecene	C_18_H_36_	252.47	0.86

^a^ Rt, retention time (min). ^b^ Compounds are listed in the order of their elution.

**Table 6:** GC–MS analysis of bacterial supernatant of the avirulent strain of *Ralstonia solanacearum*.

No.	Rt ^a^	Compound ^b^	Molecular Formula	Molecular Weight	Peak Area (%)
1	6.16	2(3H)-Furanone, 5-methyl-	C_5_H_6_O_2_	98.09	11.30
2	7.68	Butanal, 2-methyl-	C_5_H_10_O	86.13	6.12
3	9.25	Furaneol	C_6_H_8_O_3_	128.12	0.49
4	10.26	Glutaraldehyde	C_5_H_8_O_2_	100.11	1.70
5	11.64	1,3-Dioxolane, 2,4,5-trimethyl-	C_6_H_12_O_2_	116.15	18.03
6	12.22	L-1,2,3,4-Butanetetraol	C_4_H_10_O_4_	122.11	0.22
7	13.96	5-Hydroxymethylfurfural	C_6_H_6_O_3_	126.11	2.89
8	14.41	1,2,3-Propanetriol, 1-acetate	C_5_H_10_O_4_	134.13	7.32
9	20.08	2-Hexanol, acetate	C_8_H_16_O_2_	144.21	1.00
10	28.93	Cyclodecanone	C_10_H_18_O	154.24	0.50
11	30.86	n-Hexadecanoic acid (Palmitic acid)	C_16_H_32_O_2_	256.42	4.18

^a^ Rt, retention time (min). ^b^ Compounds are listed in the order of their elution.

**Table 7:** GC–MS analysis of boiled supernatant of the virulent strain of *Ralstonia solanacearum*.

No.	Rt ^a^	Compound ^b^	Molecular Formula	Molecular Weight	Peak Area (%)
1	3.70	Isobutyric acid, methyl ester	C_5_H_10_O_2_	102.13	4.77
2	5.57	Oxydiacetic acid	C_4_H_6_O_5_	134.08	0.11
3	5.73	Propanedioic acid	C_3_H_4_O_4_	104.06	0.27
4	5.91	2-Butanone, 4-hydroxy-3-methyl-	C_5_H_10_O_2_	102.13	0.26
5	5.99	Dihydroxyacetone	C_3_H_6_O_3_	90.07	1.39
6	6.09	2-Cyclopenten-1-one, 2-hydroxy-	C_5_H_6_O_2_	98.09	5.76
7	7.67	2-Hydroxy-gamma-butyrolactone	C_4_H_6_O_3_	102.08	21.17
8	8.46	1,2-Cyclopentanedione, 3-methyl-	C_6_H_8_O_2_	112.12	0.48
9	9.25	Furaneol	C_6_H_8_O_3_	128.12	3.97
10	10.22	Glutaric acid dialdehyde	C_5_H_8_O_2_	100.11	4.75
11	11.61	4H-Pyran-4-one, 2,3-dihydro-3,5-dihydroxy-6-methyl-	C_6_H_8_O_4_	144.12	16.59
12	12.96	2-Butanone, 4-hydroxy-3-methyl-	C_5_H_10_O_2_	102.13	2.04
13	13.31	1,2-Benzenediol	C_6_H_6_O_2_	110.11	1.34
14	13.97	5-Hydroxymethylfurfural	C_6_H_6_O_3_	126.11	0.87
15	14.43	1,2,3-Propanetriol, 1-acetate	C_5_H_10_O_4_	134.13	7.18
16	14.86	4H-Pyran-4-one, 2,6-dimethyl-	C_7_H_8_O_2_	124.13	1.77
17	15.82	Ethyl acetoacetate	C_6_H_10_O_3_	130.14	2.40
18	20.41	Ethanol, 2-(2-butoxyethoxy)-, acetate	C_10_H_20_O_4_	204.26	0.77
19	26.97	Cyclohexanecarboxylic acid, ethenyl ester	C_9_H_14_O_2_	154.20	2.38
20	30.45	2-Acetyl-1,3-cyclohexanedione	C_8_H_10_O_3_	154.16	1.02

^a^ Rt, retention time (min). ^b^ Compounds are listed in the order of their elution.

**Table 8:** GC–MS analysis of boiled supernatant of the avirulent strain of *Ralstonia solanacearum*.

No.	Rt ^a^	Compound ^b^	Molecular Formula	Molecular Weight	Peak Area (%)
1	3.85	2,3-Butanediol	C_4_H_10_O_2_	90.12	15.22
2	5.80	Propanedioic acid	C_3_H_4_O_4_	104.06	0.19
3	7.70	2-Hydroxy-gamma-butyrolactone	C_4_H_6_O_3_	102.08	27.78
4	9.27	Furaneol	C_6_H_8_O_3_	128.12	3.02
5	10.25	Cyclobutanol	C_4_H_8_O	72.10	4.56
6	11.62	4H-Pyran-4-one, 2,3-dihydro-3,5-dihydroxy-6-methyl-	C_6_H_8_O_4_	144.12	8.83
7	13.40	1,2-Benzenediol	C_6_H_6_O_2_	110.11	3.44
8	14.46	Propanal, 2,3-dihydroxy-, (S)-	C_3_H_6_O_3_	90.07	0.78
9	14.87	1,2-Benzenediol, 3-methyl-	C_7_H_8_O_2_	124.13	2.01
10	20.54	Pentanoic acid, 4-methyl-3-oxo-, ethyl ester	C_8_H_14_O_3_	158.19	1.71
11	35.67	Mono(2-ethylhexyl) phthalate	C_16_H_22_O_4_	278.34	1.27
12	38.81	Phthalic acid, monocyclohexyl ester	C_14_H_16_O_4_	248.27	4.59
13	39.96	1,2-Benzenedicarboxylic acid, diisooctyl ester	C_24_H_38_O_4_	390.55	1.29

^a^ Rt, retention time (min). ^b^ Compounds are listed in the order of their elution.

## Data Availability

The relevant data applicable to this research are within the paper.

## References

[1] Mansfield J., Genin S., Magori S., Citovsky V., Sriariyanum M., Ronald P., Dow M., Verdier V., Beer S.V., Machado M.A. (2012). Top 10 plant pathogenic bacteria in molecular plant pathology. Mol. Plant Pathol..

[2] Xue H., Lozano-Durán R., Macho A.P. (2020). Insights into the root invasion by the plant pathogenic bacterium *Ralstonia solanacearum*. Plants.

[3] Muthoni J., Kabira J., Shimelis H., Melis R. (2014). Spread of bacterial wilt disease of potatoes in Kenya: Who is to blame?. Int. J. Hortic..

[4] Stansbury C., McKirdy S., Mackie A., Power G. (2001). Bacterial Wilt Ralstonia Solanacearum-Race 3, Exotic Threat to Western Australia.

[5] Khairy A.M., Tohamy M.R.A., Zayed M.A., Ali M.A.S. (2021). Detecting pathogenic bacterial wilt disease of potato using biochemical markers and evaluate resistant in some cultivars. Saudi J. Biol. Sci..

[6] Balamurugan A., Sakthivel K., Gautam R.K., Sharma S.K., Kumar A., Sharma S.K., Singh U.B., Sahu P.K., Singh H.V., Sharma P.K. (2020). Ralstonia solanacearum: Biology and its management in solanaceous vegetable crops. Rhizosphere Microbes. Microorganisms for Sustainability.

[7] He D.C., He M.H., Amalin D.M., Liu W., Alvindia D.G., Zhan J. (2021). Biological control of plant diseases: An evolutionary and Eco-economic consideration. Pathogens.

[8] Yuliar, Asi Nion Y., Toyota K. (2015). Recent trends in control methods for bacterial wilt diseases caused by *Ralstonia solanacearum*. Microbes Environ..

[9] Grimault V., Prior P. (1994). Invasiveness of avirulent strains of *Pseudomonas solanacearum* in tomato cultivars, resistant or susceptible to bacterial wilt. J. Phytopathol..

[10] Arwiyanto T., Goto M., Tsuyumu S.I., Takikawa Y. (1994). Biological control of bacterial wilt of tomato by an avirulent strain of *Pseudomonas solanacearum* isolated from strelitzia reginae. Ann. Phytopathol. Soc. Jpn..

[11] Chen G., Zhou B., Zhou D., Xiao C., Ma G., Dong G. (2015). Control effects of avirulent strain Tbw1-7-3 of *Ralstonia solanacearum* on to bacterial wilt. Tobacco Sci. Technol..

[12] Trigalet A., Trigalet-Demery D. (1990). Use of avirulent mutants of *Pseudomonas solanacearum* for the biological control of bacterial wilt of tomato plants. Physiol. Mol. Plant Pathol..

[13] Zheng X., Zhu Y., Wang J., Wang Z., Liu B. (2019). Combined use of a microbial restoration substrate and avirulent *Ralstonia solanacearum* for the control of tomato bacterial wilt. Sci. Rep..

[14] Zheng X., Liu B., Zhu Y., Lin K., Ge C., Chen D. (2018). Control effects of plant vaccine avirulent *Ralstonia solanacearum* against tomato bacterial wilt disease in the field. J. Plant Prot..

[15] Milling A., Babujee L., Allen C. (2011). *Ralstonia solanacearum* extracellular polysaccharide is a specific elicitor of defense responses in wilt-resistant tomato plants. PLoS ONE.

[16] Meng F. (2013). The virulence factors of the bacterial wilt pathogen *Ralstonia solanacearum*. J. Plant Pathol. Microbiol..

[17] Moussa Z., El-Hersh M.S., El-Khateeb A.Y. (2017). Induction of potato resistance against bacterial wilt disease using *Saccharomyces cerevisiae*. Biotechnology.

[18] Sahu P.K., Singh S., Gupta A., Singh U.B., Paul S., Paul D., Kuppusamy P., Singh H.V., Saxena A.K. (2020). A Simplified protocol for reversing phenotypic conversion of *Ralstonia solanacearum* during experimentation. Int. J. Environ. Res. Public Health.

[19] Cebrián G., Condón S., Mañas P. (2017). Physiology of the inactivation of vegetative bacteria by thermal treatments: Mode of action, influence of environmental factors and inactivation kinetics. Foods.

[20] Guo C., Xie Y.J., Zhu M.T., Xiong Q., Chen Y., Yu Q., Xie J.H. (2020). Influence of different cooking methods on the nutritional and potentially harmful components of peanuts. Food Chem..

[21] Yin Q., Mu H., Zeng M., Gao D., Qin F., Chen J., He Z. (2019). Effects of heating on the total phenolic content, antioxidant activities and main functional components of simulated Chinese herb candy during boiling process. J. Food Meas. Charact..

[22] Kelman A. (1954). The relationship of pathogenicity of *Pseudomonas solanacearum* to colony appearance in a tetrazolium medium. Phytopathology.

[23] Klement Z., Rudolph K., Sands D.C. (1990). Methods in Phytobacteriology.

[24] Lelliott R.A., Stead D.E. (1987). Methods for the diagnosis of bacterial diseases of plants. Methods in Plant Pathology.

[25] Cheng C.H., Lo Y.H., Liang S.S., Ti S.C., Lin F.M., Yeh C.H., Huang H.Y., Wang T.F. (2006). SUMO modifications control assembly of synaptonemal complex and polycomplex in meiosis of *Saccharomyces cerevisiae*. Genes Dev..

[26] Wullings B.A., van Beuningen A.R., Janse J.D., Akkermans A.D.L. (1998). Detection of *Ralstonia solanacearum*, which causes brown rot of potato, by fluorescent in situ hybridization with 23S RRNA-Targeted probes. Appl. Environ. Microbiol..

[27] Kathiresan K., Manivannan S. (2006). Amylase production by *Penicillium fellutanum* isolated from mangrove rhizosphere soil. Afr. J. Biotechnol..

[28] Bailey M.J., Biely P., Poutanen K. (1992). Interlaboratory testing of methods for assay of xylanase activity. J. Biotechnol..

[29] Saber W.I.A., El-Naggar N.E.A., Abdal-Aziz S.A. (2010). Bioconversion of lignocellulosic wastes into organic acids by cellulolytic rock phosphate-solubilizing fungal isolates grown under solid-state fermentation conditions. Res. J. Microbiol..

[30] Bai Z.H., Zhang H.X., Qi H.Y., Peng X.W., Li B.J. (2004). Pectinase production by *Aspergillus niger* using wastewater in solid state fermentation for eliciting plant disease resistance. Bioresour. Technol..

[31] Al-Askar A.A., Ghoneem K.M., Hafez E.E., Saber W.I.A. (2022). A Case Study in Saudi Arabia: Biodiversity of maize seed-borne pathogenic fungi in relation to biochemical, physiological, and molecular characteristics. Plants.

[32] Nair C.B., Anith K.N. (2009). Efficacy of acibenzolar-S-methyl and rhizobacteria for the management of foliar blight disease of amaranth. J. Trop. Agric..

[33] Winstead N.N., Kelman A. (1952). Inoculation techniques for evaluating resistance to *Pseudomonas solanacearum*. Phytopathology.

[34] Malik C.P., Singh M.B. (1980). Extraction and estimation of total phenols. Plant Enzymology and Histoenzymology.

[35] Blainski A., Lopes G.C., de Mello J.C.P. (2013). Application and analysis of the folin ciocalteu method for the determination of the total phenolic content from limonium *Brasiliense* L.. Molecules.

[36] Beaudoin-Eagan L.D., Thorpe T.A. (1985). Tyrosine and phenylalanine ammonia lyase activities during shoot initiation in tobacco callus cultures. Plant Physiol..

[37] Seleim M.A., Abo-Elyousr K.A., Mohamed A.-A.A., Al-Marzoky H.A. (2014). Peroxidase and polyphenoloxidase activities as biochemical markers for biocontrol efficacy in the control of tomato bacterial wilt. J. Plant Physiol. Pathol..

[38] Kempe J., Sequeira L. (1983). Biological control of bacterial wilt of potatoes: Attempts to induce resistance by treating tubers with bacteria. Plant Dis..

[39] Bremner P.M., Taha M.A. (1966). Studies in potato agronomy. I. The effects of variety, seed size and spacing on growth, development and yield. J. Agric. Sci..

[40] Graham J. (1979). Survival of *Pseudomonas solanacearum* race 3 in plant debris and in latently infected potato tubers. Phytopathology.

[41] Moussa Z., Darwish D.B., Alrdahe S.S., Saber W.E.I.A. (2021). Innovative artificial-intelligence- based approach for the biodegradation of feather keratin by *Bacillus paramycoides*, and cytotoxicity of the resulting amino acids. Front. Microbiol..

[42] Al-Askar A.A., Rashad E.M., Ghoneem K.M., Mostafa A.A., Al-Otibi F.O., Saber W.I.A. (2021). Discovering *Penicillium polonicum* with high-lytic capacity on *Helianthus tuberosus* tubers: Oil-based preservation for mold management. Plants.

[43] Salazar S.M., Grellet C.F., Chalfoun N.R., Castagnaro A.P., Díaz Ricci J.C. (2013). Avirulent strain of colletotrichum induces a systemic resistance in strawberry. Eur. J. Plant Pathol..

[44] Patil S., Sriram S. (2020). Biological Control of Fusarium Wilt in Crop Plants Using Non-Pathogenic Isolates of Fusarium Species. Indian Phytopathol..

[45] De Lamo F.J., Spijkers S.B., Takken F.L.W. (2021). Protection to tomato wilt disease conferred by the nonpathogen *Fusarium oxysporum* Fo47 is more effective than that conferred by avirulent strains. Phytopathology.

[46] McLaughlin R.J., Sequeira L. (1988). Evaluation of an avirulent strain of *Pseudomonas solanacearum* for biological control of bacterial wilt of potato. Am. Potato J..

[47] Chen W.Y., Echandi E. (1984). Effects of avirulent bacteriocin-producing strains of *Pseudomonas solanacearum* on the control of bacterial wilt of tobacco. Plant Pathol..

[48] Verstraeten N., Braeken K., Debkumari B., Fauvart M., Fransaer J., Vermant J., Michiels J. (2008). Living on a surface: Swarming and biofilm formation. Trends Microbiol..

[49] Achari G.A., Ramesh R. (2015). Characterization of bacteria degrading 3-hydroxy palmitic acid methyl ester (3oh-pame), a quorum sensing molecule of *Ralstonia solanacearum*. Lett. Appl. Microbiol..

[50] Flavier A.B., Clough S.J., Schell M.A., Denny T.P. (1997). Identification of 3-hydroxypalmitic acid methyl ester as a novel autoregulator controlling virulence in *Ralstonia solanacearum*. Mol. Microbiol..

[51] Huang R.H., Lin W., Zhang P., Liu J.Y., Wang D., Li Y.Q., Wang X.Q., Zhang C.S., Li W., Zhao D.L. (2020). Anti-phytopathogenic bacterial metabolites from the seaweed-derived fungus *Aspergillus* sp. D40. Front. Mar. Sci..

[52] Zhang Y., Li T., Liu Y., Li X., Zhang C., Feng Z., Peng X., Li Z., Qin S., Xing K. (2019). Volatile organic compounds produced by *Pseudomonas chlororaphis* subsp. *aureofaciens* sps-41 as biological fumigants to control *Ceratocystis fimbriata* in postharvest sweet potatoes. J. Agric. Food Chem..

[53] Tuladhar P., Sasidharan S., Saudagar P. (2021). Role of phenols and polyphenols in plant defense response to biotic and abiotic stresses. Biocontrol Agents and Secondary Metabolites.

[54] Tak Y., Kumar M. (2020). Phenolics: A Key Defence secondary metabolite to counter biotic stress. Plant Phenolics in Sustainable Agriculture.

[55] Pang Z., Chen J., Wang T., Gao C., Li Z., Guo L., Xu J., Cheng Y. (2021). Linking plant secondary metabolites and plant microbiomes: A review. Front. Plant Sci..

[56] Kumar S., Abedin M.M., Singh A.K., Das S. (2020). Role of phenolic compounds in plant-defensive mechanisms. Plant Phenolics in Sustainable Agriculture.

[57] Nakahara H., Mori T., Matsuzoe N. (2021). Screening of phenotypic conversion mutant strains of *Ralstonia solanacearum* for effective biological control of *Verticillium* wilt in eggplant. Crop Prot..

[58] Ezzat A., Moussa Z. (2016). Investigating the effect of some elicitors on brown rot disease and tuber yield of potato (*Solanum tuberosum* L.). J. Prod. Dev..

[59] Bréda N.J.J. (2008). Leaf Area Index. Encyclopedia of Ecology, Five-Volume Set.

[60] Bauters L., Stojilković B., Gheysen G. (2021). Pathogens pulling the strings: Effectors manipulating salicylic acid and phenylpropanoid biosynthesis in plants. Mol. Plant Pathol..

[61] Zhang Y., Zhang W., Han L., Li J., Shi X., Hikichi Y., Ohnishi K. (2019). Involvement of a PadR regulator PrhP on virulence of *Ralstonia solanacearum* by controlling detoxification of phenolic acids and type iii secretion system. Mol. Plant Pathol..

[62] Köhl J., Kolnaar R., Ravensberg W.J. (2019). Mode of action of microbial biological control agents against plant diseases: Relevance beyond efficacy. Front. Plant Sci..

[63] Khokhani D., Tuan T., Lowe-Power T., Allen C. (2018). Plant assays for quantifying *Ralstonia solanacearum* virulence. Bio-Protocol.

[64] Prasath K.G., Tharani H., Kumar M.S., Pandian S.K. (2020). Palmitic acid inhibits the virulence factors of *Candida tropicalis*: Biofilms, cell surface hydrophobicity, ergosterol biosynthesis, and enzymatic activity. Front. Microbiol..

[65] Ma K., Kou J., Khashi U., Rahman M., Du W., Liang X., Wu F., Li W., Pan K. (2021). palmitic acid mediated change of rhizosphere and alleviation of *Fusarium* wilt disease in watermelon. Saudi J. Biol. Sci..

[66] Liu S., Ruan W., Li J., Xu H., Wang J., Gao Y., Wang J. (2008). Biological control of phytopathogenic fungi by fatty acids. Mycopathologia.

[67] Casillas-Vargas G., Ocasio-Malavé C., Medina S., Morales-Guzmán C., del Valle R.G., Carballeira N.M., Sanabria-Ríos D.J. (2021). Antibacterial fatty acids: An update of possible mechanisms of action and implications in the development of the next-generation of antibacterial agents. Prog. Lipid Res..

[68] Qiao Y., Bi J., Chen Q., Wu X., Jin X., Gou M., Yang X., Purcaro G. (2022). Rapid and sensitive quantitation of DDMP (2,3-Dihydro-3,5-Dihydroxy-6-Methyl-4H-Pyran-4-One) in baked red jujubes by HS-SPME-GC-MS/MS. Food Control.

[69] Li H., Wu C.-J., Tang X.-Y., Yu S.-J. (2019). Insights into the regulation effects of certain phenolic acids on 2,3-Dihydro-3,5-Dihydroxy-6-Methyl-4(*H*)-Pyran-4-One formation in a microaqueous glucose–proline system. J. Agric. Food Chem..

[70] Elbanhawy A.A., Elsherbiny E.A., Abd El-Mageed A.E., Abdel-Fattah G.M. (2019). Potential of fungal metabolites as a biocontrol agent against cotton aphid, *Aphis gossypii* Glover and the possible mechanisms of action. Pestic. Biochem. Physiol..

[71] Feng X., Pan L., Wang Q., Liao Z., Wang X., Zhang X., Guo W., Hu E., Li J., Xu J. (2020). Nutritional and physicochemical characteristics of purple sweet corn juice before and after boiling. PLoS ONE.

